# Supplementary material for: Pan-Tissue and -Cancer Analysis of ROR1 and ROR2 Transcript Variants Identify Novel Functional Significance for an Alternative Splice Variant of ROR1
Source: Biomedicines. 2022 Oct 13;10(10):2559. doi: 10.3390/biomedicines10102559 (PMC9599429; doi:10.3390/biomedicines10102559)
Supplement: Supplementary file 1 [file biomedicines-10-02559-s001.zip › biomedicines-1917791-supplementary.pdf]

Supplementary

Table S1. cDNA sequence for ROR1 transcripts: Multiple sequence alignment of full-length cDNA sequence of ROR1 transcript variants ROR1-v1 (ENST00000371079.6), ROR1-v2 (ENST00000371080.5) and ROR1-v3 (ENST00000545203.2). Alternating exons are represented in black and blue. Coding sequence is represented in bold text. Start codons of ROR1-v1 and ROR1-v2 are represented in red. The first five ATG codons of ROR1-v3 are highlighted in yellow and alternating codons are underscored.

```
ROR1-v2      -----GAGCGAGAGAGGGAGCGTGGAGAGCTGGAGCAGCCGCCACCGCCGCCGCCGAGGG   55
ROR1-v1      AAGTTGAGCGAGAGAGGGAGCGTGGAGAGCTGGAGCAGCCGCCACCGCCGCCGCCGAGGG   60
ROR1-v3      ------------------------------------------------------------   0

ROR1-v2      AGCCCCGGGACGGCAGCCCCTGGGCGCAGGGTGCGCTGTTCTCGGAGTCCGACCCAGGGC   115
ROR1-v1      AGCCCCGGGACGGCAGCCCCTGGGCGCAGGGTGCGCTGTTCTCGGAGTCCGACCCAGGGC   120
ROR1-v3      ------------------------------------------------------------   0

ROR1-v2      GACTCACGCCCACTGGTGCGACCCGGACAGCCTGGGACTGACCCGCCGGCCCAGGCGAGG   175
ROR1-v1      GACTCACGCCCACTGGTGCGACCCGGACAGCCTGGGACTGACCCGCCGGCCCAGGCGAGG   180
ROR1-v3      ------------------------------------------------------------   0

ROR1-v2      CTGCAGCCAGAGGGCTGGGAAGGGATCGCGCTCGCGGCATCCAGAGGCGGCCAGGCGGAG   235
ROR1-v1      CTGCAGCCAGAGGGCTGGGAAGGGATCGCGCTCGCGGCATCCAGAGGCGGCCAGGCGGAG   240
ROR1-v3      ------------------------------------------------------------   0

ROR1-v2      GCGAGGGAGCAGGTTAGAGGGACAAAGAGCTTTGCAGACGTCCCCGGCGTCCTGCGAGCG   295
ROR1-v1      GCGAGGGAGCAGGTTAGAGGGACAAAGAGCTTTGCAGACGTCCCCGGCGTCCTGCGAGCG   300
ROR1-v3      ------------------------------------------------------------   0

ROR1-v2      CCAGCGGCCGGGACGAGGCGGCCGGGAGCCCGGGAAGAGCCCGTGGATGTTCTGCGCGCG   355
ROR1-v1      CCAGCGGCCGGGACGAGGCGGCCGGGAGCCCGGGAAGAGCCCGTGGATGTTCTGCGCGCG   360
ROR1-v3      ------------------------------------------------------------   0

ROR1-v2      GCCTGGGAGCCGCCGCCGCCGCCGCCTCAGCGAGAGGAGGAATGCACCGGCCGCGCCGCC   415
ROR1-v1      GCCTGGGAGCCGCCGCCGCCGCCGCCTCAGCGAGAGGAGGAATGCACCGGCCGCGCCGCC   420
ROR1-v3      ------------------------------------------------------------   0

ROR1-v2      GCGGGACGCGCCCGCCGCTCCTGGCGCTGCTGGCCGCGCTGCTGCTGGCCGCACGCGGGG   475
ROR1-v1      GCGGGACGCGCCCGCCGCTCCTGGCGCTGCTGGCCGCGCTGCTGCTGGCCGCACGCGGGG   480
ROR1-v3      ------------------------------------------------------------   0

ROR1-v2      CTGCTGCCCAAGAAACAGAGCTGTCAGTCAGTGCTGAATTAGTGCCTACCTCATCATGGA   535
ROR1-v1      CTGCTGCCCAAGAAACAGAGCTGTCAGTCAGTGCTGAATTAGTGCCTACCTCATCATGGA   540
ROR1-v3      ----------AGAAACAGAGCTGTCAGTCAGTGCTGAATTAGTGCCTACCTCATCATGGA   50
                       **************************************************

ROR1-v2      ACATCTCAAGTGAACTCAACAAAGATTCTTACCTGACCCTCGATGAACCAATGAATAACA   595
ROR1-v1      ACATCTCAAGTGAACTCAACAAAGATTCTTACCTGACCCTCGATGAACCAATGAATAACA   600
ROR1-v3      ACATCTCAAGTGAACTCAACAAAGATTCTTACCTGACCCTCGATGAACCAATGAATAACA   110
             ************************************************************

ROR1-v2      TCACCACGTCTCTGGGCCAGACAGCAGAACTGCACTGCAAAGTCTCTGGGAATCCACCTC   655
ROR1-v1      TCACCACGTCTCTGGGCCAGACAGCAGAACTGCACTGCAAAGTCTCTGGGAATCCACCTC   660
ROR1-v3      TCACCACGTCTCTGGGCCAGACAGCAGAACTGCACTGCAAAGTCTCTGGGAATCCACCTC   170
             ************************************************************

ROR1-v2      CCACCATCCGCTGGTTCAAAAATGATGCTCCTGTGGTCCAGGAGCCCCGGAGGCTCTCCT   715
ROR1-v1      CCACCATCCGCTGGTTCAAAAATGATGCTCCTGTGGTCCAGGAGCCCCGGAGGCTCTCCT   720
```

```
ROR1-v3      CCACCATCCGCTGGTTCAAAAATGATGCTCCTGTGGTCCAGGAGCCCCGGAGGCTCTCCT    230
             ************************************************************

ROR1-v2      TTCGGTCCACCATCTATGGCTCTCGGCTGCGGATTAGAAACCTCGACACCACAGACACAG    775
ROR1-v1      TTCGGTCCACCATCTATGGCTCTCGGCTGCGGATTAGAAACCTCGACACCACAGACACAG    780
ROR1-v3      TTCGGTCCACCATCTATGGCTCTCGGCTGCGGATTAGAAACCTCGACACCACAGACACAG    290
             ************************************************************

ROR1-v2      GCTACTTCCAGTGCGTGGCAACAAACGGCAAGGAGGTGGTTTCTTCCACTGGAGTCTTGT    835
ROR1-v1      GCTACTTCCAGTGCGTGGCAACAAACGGCAAGGAGGTGGTTTCTTCCACTGGAGTCTTGT    840
ROR1-v3      GCTACTTCCAGTGCGTGGCAACAAACGGCAAGGAGGTGGTTTCTTCCACTGGAGTCTTGT    350
             ************************************************************

ROR1-v2      TTGTCAAGTTTGGCCCCCCTCCCACTGCAAGTCCAGGATACTCAGATGAGTATGAAGAAG    895
ROR1-v1      TTGTCAAGTTTGGCCCCCCTCCCACTGCAAGTCCAGGATACTCAGATGAGTATGAAGAAG    900
ROR1-v3      TTGTCAAGTTTGGCCCCCCTCCCACTGCAAGTCCAGGATACTCAGATGAGTATGAAGAAG    410
             ************************************************************

ROR1-v2      ATGGATTCTGTCAGCCATACAGAGGGATTGCATGTGCAAGATTTATTGGCAACCGCACCG    955
ROR1-v1      ATGGATTCTGTCAGCCATACAGAGGGATTGCATGTGCAAGATTTATTGGCAACCGCACCG    960
ROR1-v3      ATGGATTCTGTCAGCCATACAGAGGGATTGCATGTGCAAGATTTATTGGCAACCGCACCG    470
             ************************************************************

ROR1-v2      TCTATATGGAGTCTTTGCACATGCAAGGGGAAATAGAAAATCAGATCACAGCTGCCTTCA    1015
ROR1-v1      TCTATATGGAGTCTTTGCACATGCAAGGGGAAATAGAAAATCAGATCACAGCTGCCTTCA    1020
ROR1-v3      TCTATATGGAGTCTTTGCACATGCAAGGGGAAATAGAAAATCAGATCACAGCTGCCTTCA    530
             ************************************************************

ROR1-v2      CTATGATTGGCACTTCCAGTCACTTATCTGATAAGTGTTCTCAGTTCGCCATTCCTTCCC    1075
ROR1-v1      CTATGATTGGCACTTCCAGTCACTTATCTGATAAGTGTTCTCAGTTCGCCATTCCTTCCC    1080
ROR1-v3      CTATGATTGGCACTTCCAGTCACTTATCTGATAAGTGTTCTCAGTTCGCCATTCCTTCCC    590
             ************************************************************

ROR1-v2      TGTGCCACTATGCCTTCCCGTACTGCGATGAAACTTCATCCGTCCCAAAGCCCCGTGACT    1135
ROR1-v1      TGTGCCACTATGCCTTCCCGTACTGCGATGAAACTTCATCCGTCCCAAAGCCCCGTGACT    1140
ROR1-v3      TGTGCCACTATGCCTTCCCGTACTGCGATGAAACTTCATCCGTCCCAAAGCCCCGTGACT    650
             ************************************************************

ROR1-v2      TGTGTCGCGATGAATGTGAAATCCTGGAGAATGTCCTGTGTCAAACAGAGTACATTTTTG    1195
ROR1-v1      TGTGTCGCGATGAATGTGAAATCCTGGAGAATGTCCTGTGTCAAACAGAGTACATTTTTG    1200
ROR1-v3      TGTGTCGCGATGAATGTGAAATCCTGGAGAATGTCCTGTGTCAAACAGAGTACATTTTTG    710
             ************************************************************

ROR1-v2      CAAGATCAAATCCCATGATTCTGATGAGGCTGAAACTGCCAAACTGTGAAGATCTCCCCC    1255
ROR1-v1      CAAGATCAAATCCCATGATTCTGATGAGGCTGAAACTGCCAAACTGTGAAGATCTCCCCC    1260
ROR1-v3      CAAGATCAAATCCCATGATTCTGATGAGGCTGAAACTGCCAAACTGTGAAGATCTCCCCC    770
             ************************************************************

ROR1-v2      AGCCAGAGAGCCCAGAAGCTGCGAACTGTATCCGGATTGGAATTCCCATGGCAGATCCTA    1315
ROR1-v1      AGCCAGAGAGCCCAGAAGCTGCGAACTGTATCCGGATTGGAATTCCCATGGCAGATCCTA    1320
ROR1-v3      AGCCAGAGAGCCCAGAAGCTGCGAACTGTATCCGGATTGGAATTCCCATGGCAGATCCTA    830
             ************************************************************

ROR1-v2      TAAATAAAAATCACAAGTGTTATAACAGCACAGGTGTGGACTACCGGGGGACCGTCAGTG    1375
ROR1-v1      TAAATAAAAATCACAAGTGTTATAACAGCACAGGTGTGGACTACCGGGGGACCGTCAGTG    1380
ROR1-v3      TAAATAAAAATCACAAGTGTTATAACAGCACAGGTGTGGACTACCGGGGGACCGTCAGTG    890
             ************************************************************

ROR1-v2      TGACCAAATCAGGGCGCCAGTGCCAGCCATGGAATTCCCAGTATCCCCACACACACACTT    1435
ROR1-v1      TGACCAAATCAGGGCGCCAGTGCCAGCCATGGAATTCCCAGTATCCCCACACACACACTT    1440
ROR1-v3      TGACCAAATCAGGGCGCCAGTGCCAGCCATGGAATTCCCAGTATCCCCACACACACACTT    950
             ************************************************************

ROR1-v2      TCACCGCCCTTCGTTTCCCAGAGCTGAATGGAGGCCATTCCTACTGCCGCAACCCAGGGA    1495
ROR1-v1      TCACCGCCCTTCGTTTCCCAGAGCTGAATGGAGGCCATTCCTACTGCCGCAACCCAGGGA    1500
ROR1-v3      TCACCGCCCTTCGTTTCCCAGAGCTGAATGGAGGCCATTCCTACTGCCGCAACCCAGGGA    1010
             ************************************************************
```

```
ROR1-v2       ATCAAAAGGAAGCTCCCTGGTGCTTCACCTTGGATGAAAACTTTAAGTCTGATCTGTGTG    1555
ROR1-v1       ATCAAAAGGAAGCTCCCTGGTGCTTCACCTTGGATGAAAACTTTAAGTCTGATCTGTGTG    1560
ROR1-v3       ATCAAAAGGAAGCTCCCTGGTGCTTCACCTTGGATGAAAACTTTAAGTCTGATCTGTGTG    1070
              ************************************************************

ROR1-v2       ACATCCCAGCGTGCGGTAAATAGAAGTCATTG----------------------------    1587
ROR1-v1       ACATCCCAGCGTGCGATTCAAAGGATTCCAAGGAGAAGAATAAAATGGAAATCCTGTACA    1620
ROR1-v3       ACATCCCAGCGTGCGATTCAAAGGATTCCAAGGAGAAGAATAAAATGGAAATCCTGTACA    1130
              *************** *  * ** * **   *

ROR1-v2       ------CCCCTAATGTATTCAATCATCTTTAAAGATCCCTATCCTACCCCTCTTATTTA-    1640
ROR1-v1       TACTAGTGCCAAGTGTGGCCATTCCCCTGG--CCATTGCTT---TACTCTTCTTCTTCAT    1675
ROR1-v3       TACTAGTGCCAAGTGTGGCCATTCCCCTGG--CCATTGCTT---TACTCTTCTTCTTCAT    1185
                      ** * ***   ** **  **      **  **    *** * **** ** *

ROR1-v2       -----------GGA-------GAATCC-T----------ATAAGGGGGGCAAAGA-----    1666
ROR1-v1       TTGCGTCTGTCGGAATAACCAGAAGTCATCGTCGGCACCAGTCCAGAGGCAACCAAAACA    1735
ROR1-v3       TTGCGTCTGTCGGAATAACCAGAAGTCATCGTCGGCACCAGTCCAGAGGCAACCAAAACA    1245
                         ***       ***  * *          *     * *****  *

ROR1-v2       ------------AAATGGACAGT---ATTTGCTTG----AT-------------------    1688
ROR1-v1       CGTCAGAGGTCAAAATGTAGAGATGTCAATGCTGAATGCATATAAACCCAAGAGCAAGGC    1795
ROR1-v3       CGTCAGAGGTCAAAATGTAGAGATGTCAATGCTGAATGCATATAAACCCAAGAGCAAGGC    1305
                          ***** * **       ****      **

ROR1-v2       -----------CTCAATCTG------GTTTTAGGGTAAACCTT---------GCCGTTTC    1722
ROR1-v1       TAAAGAGCTACCTCTTTCTGCTGTACGCTTTATGGAAGAATTGGGTGAGTGTGCCTTTGG    1855
ROR1-v3       TAAAGAGCTACCTCTTTCTGCTGTACGCTTTATGGAAGAATTGGGTGAGTGTGCCTTTGG    1365
                         ***  ****      * **** ** * *  *          *** **

ROR1-v2       -----TACATAAAACACCTCGTA-----------AGGTACCAA----AACA---CGTTCT    1759
ROR1-v1       AAAAATCTATAAAGGCCATCTCTATCTCCCAGGCATGGACCATGCTCAGCTGGTTGCTAT    1915
ROR1-v3       AAAAATCTATAAAGGCCATCTCTATCTCCCAGGCATGGACCATGCTCAGCTGGTTGCTAT    1425
                   *  *****   * **              * * ****     * *     * * *

ROR1-v2       CAAGAAGTCAACTGCCTTTA--TACCTGCAGCCATTGCACTCATGGATGTAACAGGGACC    1817
ROR1-v1       CAAGACCTTGAAAGACTATAACAACCCCCAGCAATGGACGGAAT---TTCAACAAGAAGC    1972
ROR1-v3       CAAGACCTTGAAAGACTATAACAACCCCCAGCAATGGACGGAAT---TTCAACAAGAAGC    1482
              *****  *  *  * ** **   ***  **** ** *     **   *  **** * * *

ROR1-v2       C----------------------AGCC-------------CTT-CAG------------A    1829
ROR1-v1       CTCCCTAATGGCAGAACTGCACCACCCCAATATTGTCTGCCTTCTAGGTGCCGTCACTCA    2032
ROR1-v3       CTCCCTAATGGCAGAACTGCACCACCCCAATATTGTCTGCCTTCTAGGTGCCGTCACTCA    1542
              *                      * **             ***  **            *

ROR1-v2       GGCACA----------------GTTGAGACAGTTTATCACATTGATTTTTAT--------    1865
ROR1-v1       GGAACAACCTGTGTGCATGCTTTTTGAGTATATTAATCAGGGGGATCTCCATGAGTTCCT    2092
ROR1-v3       GGAACAACCTGTGTGCATGCTTTTTGAGTATATTAATCAGGGGGATCTCCATGAGTTCCT    1602
              ** ***                 *****    ** ****    *** *  **

ROR1-v2       ---------------AGAAAAAGATGTTAC-------------CCAGAATGGTCTGC---    1894
ROR1-v1       CATCATGAGATCCCCACACTCTGATGTTGGCTGCAGCAGTGATGAAGATGGGACTGTGAA    2152
ROR1-v3       CATCATGAGATCCCCACACTCTGATGTTGGCTGCAGCAGTGATGAAGATGGGACTGTGAA    1662
                             * *    ******                 ***  ** ***

ROR1-v2       ----------GTCCAAGTGGACCTTTTCAGCAAA--AAAAGGAATATTGGAAGCAGGAAG    1942
ROR1-v1       ATCCAGCCTGGACCA--CGGAGATTTTCTGCACATTGCAATTCAGATTGCAGCTGGCATG    2210
ROR1-v3       ATCCAGCCTGGACCA--CGGAGATTTTCTGCACATTGCAATTCAGATTGCAGCTGGCATG    1720
                        * ***   ***  ***** *** *    **   * **** *    * * *

ROR1-v2       AAATTGTTTTCT---GTATGCCTTAAGAACACCACAAGGCAGGATGAATCTACAAC--CA    1997
ROR1-v1       GAATACCTGTCTAGTCACTTCTTTG-----TCCACAAGGA-CCTTGCAGCTCGCAATATT    2264
ROR1-v3       GAATACCTGTCTAGTCACTTCTTTG-----TCCACAAGGA-CCTTGCAGCTCGCAATATT    1774
               ***   * ***      * * **       ********     ** * **   *

ROR1-v2       TTACTCGGTCA----------------------------------TCCAGGACAATCTG-    2022
ROR1-v1       TTAATCGGAGAGCAACTTCATGTAAAGATTTCAGACTTGGGGCTTTCCAGAGAAATTTAC    2324
ROR1-v3       TTAATCGGAGAGCAACTTCATGTAAAGATTTCAGACTTGGGGCTTTCCAGAGAAATTTAC    1834
```

```
                *** ****  *                                  *****   *** *

ROR1-v2         ----TGGGT-A-AACTGTGTCCTTCGT----TATGTCTGTTAATACTGCAGAAGAAGCAT   2072
ROR1-v1         TCCGCTGATTACTACAGGGTCCAGAGTAAGTCCTTGCTGCCCATTCGCTGGATGCCCCCT   2384
ROR1-v3         TCCGCTGATTACTACAGGGTCCAGAGTAAGTCCTTGCTGCCCATTCGCTGGATGCCCCCT   1894
                      * * *  ** * ****   **      *  ***   ** *    ** *   * *

ROR1-v2         ATAGGTATCTAGTAAGAAAATGGAATTCCTGAGTCAGTTAACTGTTCCTTTT--------   2124
ROR1-v1         GAAGCCATCATGTATGGCAAATTCTCTTCTGATTCAGATATCTGGTCCTTTGGGGTTGTC   2444
ROR1-v3         GAAGCCATCATGTATGGCAAATTCTCTTCTGATTCAGATATCTGGTCCTTTGGGGTTGTC   1954
                  **  ***  *** *  **      * **** **** ** *** ******

ROR1-v2         -TCTAGAAAATGT------TTGGA----------------------------GAAAATA   2148
ROR1-v1         TTGTGGGAGATTTTCAGTTTTGGACTCCAGCCATATTATGGATTCAGTAACCAGGAAGTG   2504
ROR1-v3         TTGTGGGAGATTTTCAGTTTTGGACTCCAGCCATATTATGGATTCAGTAACCAGGAAGTG   2014
                 * * * * ** *      *****                             * ** *

ROR1-v2         ATGAAAATGGGCCAAGCATGGTGGCTTATACCTGTAATCCCAACACT-------------   2195
ROR1-v1         ATTGAGATGGTGAGAAAACGGCAGCTCTTACCA-TGCTCTGAAGACTGCCCACCCAGAAT   2563
ROR1-v3         ATTGAGATGGTGAGAAAACGGCAGCTCTTACCA-TGCTCTGAAGACTGCCCACCCAGAAT   2073
                **  * ****    *  * **  ***  ****  *  **   ** ***

ROR1-v2         CTA---------GGAAG-GCCGAGGCAGGAGGATCATT---TGAGCCCAGGGGTTCAAGA   2242
ROR1-v1         GTACAGCCTCATGACAGAGTGCTGGAATGAGATTCCTTCTAGGAGACCAAGATTTAAAGA   2623
ROR1-v3         GTACAGCCTCATGACAGAGTGCTGGAATGAGATTCCTTCTAGGAGACCAAGATTTAAAGA   2133
                 **         *  ** *    ** * ***  ** **    *** *** *  ** ****

ROR1-v2         C---------CAGCCCAGG----------CAACATAGTGAGACTCCATCTCTACCAAAA-   2282
ROR1-v1         TATTCACGTCCGGCTTCGGTCCTGGGAGGGACTCTCAAGTCACACAAGCTCTACTACTCC   2683
ROR1-v3         TATTCACGTCCGGCTTCGGTCCTGGGAGGGACTCTCAAGTCACACAAGCTCTACTACTCC   2193
                          * **   **           *   *   *  ** * * ****** *

ROR1-v2         ----AAAAGAAAA--AAAAAAAAAGAAAA-------------------------------   2305
ROR1-v1         TTCAGGGGGAAATGCCACCACACAGACAACCTCCCTCAGTGCCAGCCCAGTGAGTAATCT   2743
ROR1-v3         TTCAGGGGGAAATGCCACCACACAGACAACCTCCCTCAGTGCCAGCCCAGTGAGTAATCT   2253
                        ****    *  * * *** **

ROR1-v2         ------------------------------------------------------------   2305
ROR1-v1         CAGTAACCCCAGATATCCTAATTACATGTTCCCGAGCCAGGGTATTACACCACAGGGCCA   2803
ROR1-v3         CAGTAACCCCAGATATCCTAATTACATGTTCCCGAGCCAGGGTATTACACCACAGGGCCA   2313

ROR1-v2         ------------------------------------------------------------   2305
ROR1-v1         GATTGCTGGTTTCATTGGCCCGCCAATACCTCAGAACCAGCGATTCATTCCCATCAATGG   2863
ROR1-v3         GATTGCTGGTTTCATTGGCCCGCCAATACCTCAGAACCAGCGATTCATTCCCATCAATGG   2373

ROR1-v2         ------------------------------------------------------------   2305
ROR1-v1         ATACCCAATACCTCCTGGATATGCAGCGTTTCCAGCTGCCCACTACCAGCCAACAGGTCC   2923
ROR1-v3         ATACCCAATACCTCCTGGATATGCAGCGTTTCCAGCTGCCCACTACCAGCCAACAGGTCC   2433

ROR1-v2         ------------------------------------------------------------   2305
ROR1-v1         TCCCAGAGTGATTCAGCACTGCCCACCTCCCAAGAGTCGGTCCCCAAGCAGTGCCAGTGG   2983
ROR1-v3         TCCCAGAGTGATTCAGCACTGCCCACCTCCCAAGAGTCGGTCCCCAAGCAGTGCCAGTGG   2493

ROR1-v2         ------------------------------------------------------------   2305
ROR1-v1         GTCGACTAGCACTGGCCATGTGACTAGCTTGCCCTCATCAGGATCCAATCAGGAAGCAAA   3043
ROR1-v3         GTCGACTAGCACTGGCCATGTGACTAGCTTGCCCTCATCAGGATCCAATCAGGAAGCAAA   2553

ROR1-v2         ------------------------------------------------------------   2305
ROR1-v1         TATTCCTTTACTACCACACATGTCAATTCCAAATCATCCTGGTGGAATGGGTATCACCGT   3103
ROR1-v3         TATTCCTTTACTACCACACATGTCAATTCCAAATCATCCTGGTGGAATGGGTATCACCGT   2613

ROR1-v2         ------------------------------------------------------------   2305
```

ROR1-v1 **TTTTGGCAACAAATCTCAAAAACCCTACAAAATTGACTCAAAGCAAGCATCTTTACTAGG** 3163
ROR1-v3 **TTTTGGCAACAAATCTCAAAAACCCTACAAAATTGACTCAAAGCAAGCATCTTTACTAGG** 2673

ROR1-v2 -- 2305
ROR1-v1 **AGACGCCAATATTCATGGACACACCGAATCTATGATTTCTGCAGAACTGTAA**AATGCACA 3223
ROR1-v3 **AGACGCCAATATTCATGGACACACCGAATCTATGATTTCTGCAGAACTGTAA**AATGCACA 2733

ROR1-v2 -- 2305
ROR1-v1 ACTTTTGTAAATGTGGTATACAGGACAAACTAGACGGCCGTAGAAAAGATTTATATTCAA 3283
ROR1-v3 ACTTTTGTAAATGTGGTATACAGGACAAACTAGACGGCCGTAGAAAAGATTTATATTCAA 2793

ROR1-v2 -- 2305
ROR1-v1 ATGTTTTTATTAAAGTAAGGTTCTCATTTAGCAGACATCGCAACAAGTACCTTCTGTGAA 3343
ROR1-v3 ATGTTTTTATTAAAGTAAGGTTCTCATTTAGCAGACATCGCAACAAGTACCTTCTGTGAA 2853

ROR1-v2 -- 2305
ROR1-v1 GTTTCACTGTGTCTTACCAAGCAGGACAGACACTCGGCCAGAAAAAAAAAAAAAAAAAAA 3403
ROR1-v3 GTTTCACTGTGTCTTACCAAGCAGGACAGACACTCGGCCAGAAAAAAAAAAAAAAAAAAA 2913

ROR1-v2 -- 2305
ROR1-v1 AAACAAGCAAACAAAAACATTGTGGGATGTGCACTCCATTGGAGTGCATGACATGGCATT 3463
ROR1-v3 AAACAAGCAAACAAAAACATTGTGGGATGTGCACTCCATTGGAGTGCATGACATGGCATT 2973

ROR1-v2 -- 2305
ROR1-v1 GGGATTGGAACATGTGGTTTCGAGCACTGAAAGCTGCAAACCAGTGAAGAGGAAAAGAAC 3523
ROR1-v3 GGGATTGGAACATGTGGTTTCGAGCACTGAAAGCTGCAAACCAGTGAAGAGGAAAAGAAC 3033

ROR1-v2 -- 2305
ROR1-v1 CTTGTGATTAAATATAAAACCAAAAGTCAAATGGTGCTTTGTGTTTTAGCCTTCAGTCAC 3583
ROR1-v3 CTTGTGATTAAATATAAAACCAAAAGTCAAATGGTGCTTTGTGTTTTAGCCTTCAGTCAC 3093

ROR1-v2 -- 2305
ROR1-v1 CATGACTGGTCTCTCCCCCAGATGTATATATACCATAGCATTTGTCTACCTGCTGTCTTT 3643
ROR1-v3 CATGACTGGTCTCTCCCCCAGATGTATATATACCATAGCATTTGTCTACCTGCTGTCTTT 3153

ROR1-v2 -- 2305
ROR1-v1 TCTTCAGGACAGATGTTCAGGAATTATATTGATTGAATTTAGACTCTGTGCATGTTCTTA 3703
ROR1-v3 TCTTCAGGACAGATGTTCAGGAATTATATTGATTGAATTTAGACTCTGTGCATGTTCTTA 3213

ROR1-v2 -- 2305
ROR1-v1 TGGAAATGATGTTCAGAATCCATGAAGAAACTTCAGGCCAAATTTGAAACCCTGGAGGGA 3763
ROR1-v3 TGGAAATGATGTTCAGAATCCATGAAGAAACTTCAGGCCAAATTTGAAACCCTGGAGGGA 3273

ROR1-v2 -- 2305
ROR1-v1 AATGAGCCATAAGGGAAGTATAACAAGCCCTGAAGCCTTTTATGTCGTTGTGCTTCTTTG 3823
ROR1-v3 AATGAGCCATAAGGGAAGTATAACAAGCCCTGAAGCCTTTTATGTCGTTGTGCTTCTTTG 3333

ROR1-v2 -- 2305
ROR1-v1 GGAAGGTGTAGAGTGTGCCTTTTTGTGAATCCTCCTCTGATCATGAGGGTCTTTCCCACA 3883
ROR1-v3 GGAAGGTGTAGAGTGTGCCTTTTTGTGAATCCTCCTCTGATCATGAGGGTCTTTCCCACA 3393

ROR1-v2 -- 2305
ROR1-v1 GTTTCTCACAGTGTGTTTACACTGCCCTTGGAATAACACAGCGCATCAGACCATAAGAAG 3943
ROR1-v3 GTTTCTCACAGTGTGTTTACACTGCCCTTGGAATAACACAGCGCATCAGACCATAAGAAG 3453

```
ROR1-v2       ------------------------------------------------------------    2305
ROR1-v1       GCTAGATGTGGATGCTAGAATTGATTGTTGGTTGATAGTTCTCTTTGCTGGATTAGGAAT    4003
ROR1-v3       GCTAGATGTGGATGCTAGAATTGATTGTTGGTTGATAGTTCTCTTTGCTGGATTAGGAAT    3513

ROR1-v2       ------------------------------------------------------------    2305
ROR1-v1       GAGGCGCCAAAGGAAGCACAGCCAGGAAAATGGCCCCACAGCCTAGATCAGCATCTGTGG    4063
ROR1-v3       GAGGCGCCAAAGGAAGCACAGCCAGGAAAATGGCCCCACAGCCTAGATCAGCATCTGTGG    3573

ROR1-v2       ------------------------------------------------------------    2305
ROR1-v1       GAAAGGAAAAAGGGTTCCCATGGGGACAGCCCCCATAGGAGTTTTCTGGAACCAGTAACA    4123
ROR1-v3       GAAAGGAAAAAGGGTTCCCATGGGGACAGCCCCCATAGGAGTTTTCTGGAACCAGTAACA    3633

ROR1-v2       ------------------------------------------------------------    2305
ROR1-v1       CTGAAAAATAAGTGTGTGGCTACAGATGAGCACGCCCACCCCTTGCAACTCCCTGTTTAC    4183
ROR1-v3       CTGAAAAATAAGTGTGTGGCTACAGATGAGCACGCCCACCCCTTGCAACTCCCTGTTTAC    3693

ROR1-v2       ------------------------------------------------------------    2305
ROR1-v1       AAGTTGTCCGAGGCATTGGAGTGCTTATGGTCAATGGGCTCTAGGGAAGTAGGAAACTCC    4243
ROR1-v3       AAGTTGTCCGAGGCATTGGAGTGCTTATGGTCAATGGGCTCTAGGGAAGTAGGAAACTCC    3753

ROR1-v2       ------------------------------------------------------------    2305
ROR1-v1       ATCATGATACAAATGTCTAGTAATTTTAAAGTTTCTTTCCCTTTTTTTCTGTGCTGGAAA    4303
ROR1-v3       ATCATGATACAAATGTCTAGTAATTTTAAAGTTTCTTTCCCTTTTTTTCTGTGCTGGAAA    3813

ROR1-v2       ------------------------------------------------------------    2305
ROR1-v1       TGTTCACAGATTTGATTCCCGCCCCAAGAATTACAACAATGTATTCATACAGTCAGTTGG    4363
ROR1-v3       TGTTCACAGATTTGATTCCCGCCCCAAGAATTACAACAATGTATTCATACAGTCAGTTGG    3873

ROR1-v2       ------------------------------------------------------------    2305
ROR1-v1       AATCCAAAGGCAATTAATTCTATTTTGCAAAATATGATGGTCTTCCTAAAAAACAAGTAC    4423
ROR1-v3       AATCCAAAGGCAATTAATTCTATTTTGCAAAATATGATGGTCTTCCTAAAAAACAAGTAC    3933

ROR1-v2       ------------------------------------------------------------    2305
ROR1-v1       TGAGTTCTCATTTCAAAAGTTACCAAGAACTGAATTCTTTAAACTAGCAAACCGAAGTAA    4483
ROR1-v3       TGAGTTCTCATTTCAAAAGTTACCAAGAACTGAATTCTTTAAACTAGCAAACCGAAGTAA    3993

ROR1-v2       ------------------------------------------------------------    2305
ROR1-v1       CATGACCATTTTTGCCTTAGTGGGAGGTTTCAAATGTGCAGCTCATGGCATTTACCTGCC    4543
ROR1-v3       CATGACCATTTTTGCCTTAGTGGGAGGTTTCAAATGTGCAGCTCATGGCATTTACCTGCC    4053

ROR1-v2       ------------------------------------------------------------    2305
ROR1-v1       GACCATCTTTTGCCAAGTTTAGAATTCTTATGCGTTTCAAGTTCTATATAGAAAGTAATT    4603
ROR1-v3       GACCATCTTTTGCCAAGTTTAGAATTCTTATGCGTTTCAAGTTCTATATAGAAAGTAATT    4113

ROR1-v2       ------------------------------------------------------------    2305
ROR1-v1       TTACTTTTGATTTTTCTCTTGTTAAAAAAAAACTCCTTTATTCTAAGAACAATGTCTCAA    4663
ROR1-v3       TTACTTTTGATTTTTCTCTTGTTAAAAAAAAACTCCTTTATTCTAAGAACAATGTCTCAA    4173

ROR1-v2       ------------------------------------------------------------    2305
ROR1-v1       AGTCTCATTTTTACTTTAAAGGTATAAGAGACTTCTAAAGAGACTTACGGGATATAAAAG    4723
ROR1-v3       AGTCTCATTTTTACTTTAAAGGTATAAGAGACTTCTAAAGAGACTTACGGGATATAAAAG    4233

ROR1-v2       ------------------------------------------------------------    2305
ROR1-v1       TAATTCCTGGAAATGATATTTGATGAGGAGAATGTAAGAGAATGAAAAACACATTGATGT    4783
```

```
ROR1-v3       TAATTCCTGGAAATGATATTTGATGAGGAGAATGTAAGAGAATGAAAAACACATTGATGT    4293

ROR1-v2       ------------------------------------------------------------    2305
ROR1-v1       TTTCATTTTTAAAAAATACAACTAGCATGAGAAATCAAGAAAATATGTTTACCAAAATGC    4843
ROR1-v3       TTTCATTTTTAAAAAATACAACTAGCATGAGAAATCAAGAAAATATGTTTACCAAAATGC    4353

ROR1-v2       ------------------------------------------------------------    2305
ROR1-v1       ATTGCAATTTTCCCAAACCTGAGTCTTCAAATAACAAACATGAACTTATAGGTACTGTGA    4903
ROR1-v3       ATTGCAATTTTCCCAAACCTGAGTCTTCAAATAACAAACATGAACTTATAGGTACTGTGA    4413

ROR1-v2       ------------------------------------------------------------    2305
ROR1-v1       ACTAGAAGAATTGGTTATATCCAGATTTCTGGGAGATAAATTAAAACATATTTTTGTGGC    4963
ROR1-v3       ACTAGAAGAATTGGTTATATCCAGATTTCTGGGAGATAAATTAAAACATATTTTTGTGGC    4473

ROR1-v2       ------------------------------------------------------------    2305
ROR1-v1       ATAAATAAGCTGATTCAGAAGTTACATTTCTTAACTTTAGGTAGAGGAGAATATTTGTAT    5023
ROR1-v3       ATAAATAAGCTGATTCAGAAGTTACATTTCTTAACTTTAGGTAGAGGAGAATATTTGTAT    4533

ROR1-v2       ------------------------------------------------------------    2305
ROR1-v1       CCTTTTGTTGCTATGCAACTGTTTAATGATGAAGCTTCAAACCACAATTTTGTATATCAT    5083
ROR1-v3       CCTTTTGTTGCTATGCAACTGTTTAATGATGAAGCTTCAAACCACAATTTTGTATATCAT    4593

ROR1-v2       ------------------------------------------------------------    2305
ROR1-v1       ATGACAATCAATTGTTTTCCAAGAATATTTATTATTTTAAGTACACAATTTCAGTGAATC    5143
ROR1-v3       ATGACAATCAATTGTTTTCCAAGAATATTTATTATTTTAAGTACACAATTTCAGTGAATC    4653

ROR1-v2       ------------------------------------------------------------    2305
ROR1-v1       TTGAGTTTTTCTAGGAGTCCCTTAAAGGGAAATTAGCATTCCATGAGCCAGTAAAATACC    5203
ROR1-v3       TTGAGTTTTTCTAGGAGTCCCTTAAAGGGAAATTAGCATTCCATGAGCCAGTAAAATACC    4713

ROR1-v2       ------------------------------------------------------------    2305
ROR1-v1       TACTCTGGTAAAACATTATTATGGTTAAAAAGTAAATTCAAGTTAGTTTTTTATAAAGAA    5263
ROR1-v3       TACTCTGGTAAAACATTATTATGGTTAAAAAGTAAATTCAAGTTAGTTTTTTATAAAGAA    4773

ROR1-v2       ------------------------------------------------------------    2305
ROR1-v1       CTATAACATTTATTTTAAACATTTTATAATAACTGAAAACATTAAAGTGAGCAAATGAAA    5323
ROR1-v3       CTATAACATTTATTTTAAACATTTTATAATAACTGAAAACATTAAAGTGAGCAAATGAAA    4833

ROR1-v2       ------------------------------------------------------------    2305
ROR1-v1       TTTCAAAACCACTTGTAATAATGTATTTTATAATCGCACTGTGATACTATATAACACAGT    5383
ROR1-v3       TTTCAAAACCACTTGTAATAATGTATTTTATAATCGCACTGTGATACTATATAACACAGT    4893

ROR1-v2       ------------------------------------------------------------    2305
ROR1-v1       CTCTTTTGTATTAAAATAGTATTTTTTTCACAAGCTGAAAAATATCTTTTCCTTTGTTGA    5443
ROR1-v3       CTCTTTTGTATTAAAATAGTATTTTTTTCACAAGCTGAAAAATATCTTTTCCTTTGTTGA    4953

ROR1-v2       ------------------------------------------------------------    2305
ROR1-v1       CAATGTTTTGTAAGATGACTTTATTTTCAGATCTTTTTCTCCTTTTTTGCACAAAACTAT    5503
ROR1-v3       CAATGTTTTGTAAGATGACTTTATTTTCAGATCTTTTTCTCCTTTTTTGCACAAAACTAT    5013

ROR1-v2       ------------------------------------------------------------    2305
ROR1-v1       GCTTATGGTTTGTGTCACAGAAGTGAAAATATATCTTTGCATTTTTATATCTGGTCTGTT    5563
ROR1-v3       GCTTATGGTTTGTGTCACAGAAGTGAAAATATATCTTTGCATTTTTATATCTGGTCTGTT    5073
```

```
ROR1-v2       ------------------------------------------------------------   2305
ROR1-v1       TTTCTTGTTTCCTTTGTTTTTAACTTGATATAGATTTTCTTCAAATATATAATGGCAATT   5623
ROR1-v3       TTTCTTGTTTCCTTTGTTTTTAACTTGATATAGATTTTCTTCAAATATATAATGGCAATT   5133

ROR1-v2       ------------------------------------------------------------   2305
ROR1-v1       TTCAGATATCTCACCTTACCATATCTTTCCTTATTTTCACTGCATGCATTTAATCACTGT   5683
ROR1-v3       TTCAGATATCTCACCTTACCATATCTTTCCTTATTTTCACTGCATGCATTTAATCACTGT   5193

ROR1-v2       ------------------------------------------------------------   2305
ROR1-v1       ATTACTTAATGTTTGATTTGTTATTATGGGCATTTCAAATAGGCAAGCATTGAATTGTAA   5743
ROR1-v3       ATTACTTAATGTTTGATTTGTTATTATGGGCATTTCAAATAGGCAAGCATTGAATTGTAA   5253

ROR1-v2       ------------------------------------------------------------   2305
ROR1-v1       TGACAAAAAGGCTATTTTATATTAAGGATATATGCATTTGTATTTCACACACCAGAGATG   5803
ROR1-v3       TGACAAAAAGGCTATTTTATATTAAGGATATATGCATTTGTATTTCACACACCAGAGATG   5313

ROR1-v2       -------------------------------------------------------        2305
ROR1-v1       ATATTAAACACTGATTATTTTATGCTGCTGTTTATTAAAAATGTTTACTATAAAA        5858
ROR1-v3       ATATTAAACACTGATTATTTTATGCTGCTGTTTATTAAAAATGTTTACT------        5362
```
